# Single versus Double Stenting in NSTEMI Patients with Complex Left Main Bifurcation Disease

**DOI:** 10.3390/jcm11123559

**Published:** 2022-06-20

**Authors:** Gianluca Rigatelli, Marco Zuin, Filippo Gianese, Dario Adami, Mauro Carraro, Loris Roncon

**Affiliations:** 1 Cardiovascular Diagnosis and Endoluminal Interventions, Department of Specialistic Medicine, Rovigo General Hospital, 45100 Rovigo, Italy; filippo.gianese@aulss5.veneto.it (F.G.); dario.adami@aulss5.veneto.it (D.A.); 2 Department of Translational Medicine, Section of Internal and Cardio-Respiratory Medicine, University of Ferrara, 44121 Ferrara, Italy; zuinml@yahoo.it; 3 Division of Cardiology, Department of Specialistic Medicine, Rovigo General Hospital, 45100 Rovigo, Italy; mauro.carraro@aulss5.veneto.it (M.C.); roncon.loris@gmail.com (L.R.)

**Keywords:** left main, bifurcation stenting, acute coronary syndrome, NSTEMI

## Abstract

**Background:** Among patients with non-ST-segment elevation myocardial infarction (NSTEMI) the presence of a bifurcation left main (LM) disease represents a particular subset graved by both clinical and technical challenges. We sought to assess the long-term outcomes of patients with NSTEMI treated either by single or double stent strategy, having an LM bifurcation culprit lesion. **Methods:** We retrospectively analyzed the procedural and medical data of consecutive patients referred to our center for NSTEMI due to complex LM bifurcation disease as the culprit lesion, treated using either single or dual stenting (provisional stenting, T or T-and-Protrusion (TAP), Culotte, and Nano-inverted-T (NIT)) techniques between January 2008 and May 2018. Target lesion failure (TLF) was defined as the composite of cardiovascular death, target-vessel myocardial infarction (MI), and clinically driven target lesion revascularization (TLR). **Results:** Four hundred and forty-five patients (54.1% males, mean age 70.3 ± 12.8 years, mean Syntax score 31.6 ± 6.3) were evaluated. Of these, 155 patients (34.8%) were treated using a single stent while the remaining were treated with a double stent strategy. After a mean follow-up of 37.1 months (*IQR* 22.1-39.3), TLF rate was 8.7% (n = 39): 5/155 (3.2%) in the crossover group; 10/53 (18.8%) in T/TAP group, 14/89 (15.7%) in the culotte group, and 10/148 (6.7%) in the NIT group of patients. Cardiovascular mortality rate was 2.9% (n = 13) while stent thrombosis was 0.89% (n = 4). On multivariate analysis dyslipidemia, Syntax score > 25, triple vessel disease, additional LM ostial, or LM body lesions and the use of Rotablator, were independent predictors of TLF. **Conclusions:** Either a single or double stent strategy resulted in low rates of TLF, cardiovascular death, and stent thrombosis in the long-term period in NSTEMI LM patients with contraindications or refusal of surgery. A single stent strategy appeared to have a slightly better outcome compared to a 2-stent strategy.

## 1. Introduction

Nowadays, acute coronary syndrome (ACS) and specifically non-ST elevation myocardial infarction (NSTEMI) often represent the initial presentation of ischemic heart disease [1]. Among patients with NSTEMI presentation, left main (LM) bifurcation disease constitutes a particular subset graved by both management and technical issues. Current guidelines recommend a Heart team discussion-based approach to this subset of patients and possible surgical revascularization [2]. Patients having a higher surgical risk and/or refusing the surgical option can be offered PCI with either a single stent or double stent strategy depending on the complexity of the bifurcation disease and the operators’ preferences/skills. The current literature suggests that clinical results of complex LM bifurcation PCI are encouraging in the general population [3,4] while reports about long-term outcomes in NSTEMI patients are still scarce. In this retrospective analysis, we sought to analyze the clinical and procedural characteristics of patients with NSTEMI presentation treated by either a single or double stent strategy and assess their long-term outcomes.

## 2. Materials and Methods

### 2.1. Population Enrolled

We retrospectively analyzed the procedural and medical data of consecutive patients referred to our center with NSTEMI presentation due to complex LM bifurcation disease as a culprit lesion having contraindications or refusing surgery after evaluation of the heart team, treated by provisional stenting, Culotte, T-and-Protrusion (TAP), and Nano-inverted-T (NIT) stenting [5] between 1 January 2008 and 1 May 2018. Classical and modified Crush and Dk-crush were not applied in our center because it is believed less respectful of bifurcation physiology in LM disease. Traditional cardiovascular risk factors, Canadian Cardiovascular Score class (CCS), EuroSCORE II [6], SYNTAX score [7], MEDINA classification [8], as well as pre-and post-procedural angiographic characteristics were revised and analyzed as mandatory inclusion criteria, by the Rovigo General Hospital Heart Team, which includes a clinical cardiologist, a cardiac surgeon, and an interventional cardiologist. Written informed consent to the indexed procedure was obtained from all patients before interventions.

Inclusion criteria for our retrospective analysis were all of the following: (1) NSTEMI presentation following the current guidelines [9]; (2) percutaneous coronary intervention (PCI) intended in a true de novo LM bifurcation lesion (Medina 1,1,1 or 0,1,1), with >50% diameter stenosis (DS) of both the ostial left anterior descending (LAD) and left circumflex (LCx) coronary arteries by visual estimation and quantitative coronary angiography (QCA) and possibly confirmed by Fractional Flow Reserve (FFR) or IVUS. Lesions presenting a TIMI < 3 or appearance of >90% stenosis were assessed eventually only by QCA and IVUS. Conversely, exclusion criteria were: (1) patients who developed an intraprocedural ST-elevation myocardial infarction (STEMI) with vessel occlusion as a complication of an elective procedure, (2) previous CABG; (3) in-stent restenosis (ISR); (4) any clinical condition that would interfere with medications compliance or long-term follow-up.

### 2.2. Definitions

Target lesion failure (TLF) was defined as the composite of cardiovascular death, target-vessel MI (TVMI), and clinically driven target lesion revascularization (TLR). TLR was defined as any repeat percutaneous intervention of the target lesion (including 5 mm proximal and 5 mm distal to the target lesion). Cardiovascular mortality from cardiac causes was defined as any death without a clear non-cardiac cause. Protocol-defined periprocedural acute myocardial infarction (AMI) was defined as a post-procedure cTn rise by > 20% with respect to the pre-procedural values. However, the absolute post-procedural value must still be at least five times the 99th percentile URL. In addition, one of the following elements is required: New ischemic ECG changes; Development of new pathological Q waves; Imaging evidence of new loss of viable myocardium or new regional wall motion abnormality in a pattern consistent with an ischemic etiology. Angiographic findings are consistent with procedural flow-limiting complication such as coronary dissection, occlusion of a major epicardial artery or a side branch occlusion/thrombus, disruption of collateral flow, or distal embolization (Type 4a MI) [10]. Spontaneous MI was defined as the detection of a rise and/or fall of cTn values with at least one value above the 99th percentile URL and with at least one of the following: Symptoms of acute myocardial ischemia; New ischemic ECG changes; Development of pathological Q waves; Imaging evidence of new loss of viable myocardium or new regional wall motion abnormality in a pattern consistent with an ischemic etiology; Identification of a coronary thrombus by angiography including intracoronary imaging or by autopsy (Type 1 MI) [10]. Both Type 4a MI related to the index LM PCI and periprocedural MI related to the ischemic-driven revascularization were included in TLF. Finally, MACE (major adverse cardiovascular event) is defined as a composite of nonfatal stroke, nonfatal myocardial infarction, and cardiovascular death.

Stent thrombosis (ST) was classified according to the Academic Research Consortium (ARC) definitions as definite, probable, or possible and as early (0–30 days), late (31–360 days), or very late (>360 days). In-stent restenosis (ISR) was evaluated by QCA and eventually FFR if the luminal narrowing was <70% and classified as focal (<10 mm long), diffuse (>10 mm long), proliferative (>10 mm long and extending outside the stent edges), or totally occluded [11].

### 2.3. Interventional Protocol and Techniques

A 6F right radial approach has been selected whenever possible. During PCI, patients were anticoagulated with unfractionated heparin (a bolus of 40 U/kg and additional heparin to achieve an activated clotting time of 250–300 s). The selection of stenting techniques was left to operator choice and included provisional stenting, Culotte, T-and-Protrusion (TAP), and Nano-inverted-T stenting. Patients could receive the Orsiro (Biotronik Inc., Bulach, Switzerland), Xience (Abbot Inc., Abbot Park, IL, USA) and Promus Premiere (Boston Scientific Inc., Marlborough, MA, USA) or the Onyx Resolute (Medtronic Inc., Galway, Ireland) stents, basing the diameter of the main vessel stent on Finet’s law [12], or preferably IVUS, which was recommended in all enrolled patients whenever possible, depending on availability. The kissing balloon was considered mandatory in all double stent techniques, whereas in provisional stenting, the preferred strategy was POT or POT-Side-POT technique. Additional significant lesions in other vessels were treated with staged procedures and a routine last generation DES of the operator’s choice. Twelve-month Ticagrelor or Prasugrel treatment or 12-month Clopidogrel 75 mg in case of excessive bleeding risk or frailty and life-long aspirin was recommended to all patients according to our regional guidelines.

### 2.4. FFR and IVUS Protocol

FFR evaluation was conducted using PressureWire X device (Abbot Medical, Plymouth, MN, USA) and intracoronary bolus injection of Adenosin with a dilution of 12 mg in 250 mL of NaCl solution (6–8 mL per run): a mean cut-off <0.79 on at least three runs was considered significant. A pressure wire was eventually placed in both LAD and LCx or in the lesions with <80% stenosis. Intravascular Ultrasound (IVUS) examination was performed routinely following current recommendations using the 3F Opticross coronary IVUS catheter (Boston Scientific, Fremont, CA, USA) and automatic pull-back system (0.5 mm/s). An on-line ultrasound assessment was performed in diastole. IVUS images were recorded after administration of 100–200 mg of nitroglycerin. A segment of 0.5 mm proximally and distally the lesion/stent was analyzed using a motorized transducer pull-back. IVUS images were interpreted by the treating physician and at least one experienced IVUS technician.

### 2.5. Follow-Up

Per institutional protocol, follow-up was conducted by physical examination and surface 12-lead electrocardiogram at 1, 6, and 12 months and then yearly. Transthoracic echocardiography (TTE) was scheduled at 6 months and then yearly. Exercise tests have been conducted at 6 months and thereafter at the referral physician’s discretion. Angiography with IVUS control was performed only at the time of additional vessel treatment or based on clinical symptoms or instrumental evidence of myocardial ischemia on exercise or nuclear stress test. Post-discharge survival status was obtained from the Municipal Civil Registries. Information on the occurrence of acute MI or repeated interventions at follow-up was collected by consulting our institutional electronic database and by contacting referring physicians and institutions and all living patients.

### 2.6. Statistical Analysis

Continuous variables were presented as mean ± standard deviation while categorical data were summarized as frequencies and relative percentages. For continuous variables, normal distribution was evaluated with the Kolmogorov–Smirnov test. Differences among groups were analyzed by the Student’s t-test or one-way analysis of variance (ANOVA) followed by the post hoc Bonferroni test. The multivariate Cox-regression analysis and Kaplan Meier curve were used to evaluate the difference in TLF among patients treated with single or double stinting techniques. A statistical significance was defined as *p* < 0.05. Statistical analyses were performed using SPSS package version 20.0 (SPSS, Chicago, IL, USA).

## 3. Results

### 3.1. Population and Procedures

Four hundred and forty-five patients (241 males, mean age 70.3 ± 12.8 years, mean Syntax score 31.6 ± 6.3) were evaluated: 155 patients (34.8%) were treated with a single stent while 65.2% with a double stent strategy. Twenty-three patients (5.1%) initially managed with the 1-stent technique were converted to the 2-stent technique during the procedure, while 267 were managed upfront with the 2-stent technique. Reasons for conversion were suboptimal LCx results after cross-over stenting in 16 patients, and a flow-limiting dissection of proximal LCX after cross-over stenting in the other 6 patients.

The clinical characteristics and comorbidities of the population enrolled are shown in Table 1 and Table 2: single and double stent strategy groups of patients were homogeneous for major clinical variables while EUROSCORE was higher in double compared to single stent strategy group of patients and in patients submitted to NIT compared to Culotte and T/TAP. Coronary angiography evidenced a mean angle between LM and LCx of 64.8 ± 20.7° (range 17 to 91 degrees). Lesion characteristics are shown in Table 3. IVUS was performed in 77.7% (n = 115/148), 32.2% (n = 50/155), 18.8% (n = 10/53), and 57.3% (n = 51/89) of patients stented using NIT, provisional stenting, T or TAP stenting and Culotte, respectively (Table 4). FFR confirmed was needed in 65 patients (14.6%).

### 3.2. PCI Immediate Outcomes

Immediate success was 100%. Six French radial access was used in the majority of patients (74.1%, 330/445) while a 7 in 6F sheath was used in 60 patients (13.5%) and a femoral 7F was used in 55 patients (12.3%). The intraprocedural complications rate was 2.2% (10/445) and included: two patients experienced stent loss, three patients had intraprocedural occlusion of LCx recanalized during the procedure, and five patients had a femoral hematoma (two requiring blood transfusion).

### 3.3. PCI Follow Up Outcomes

**Figure 1:**
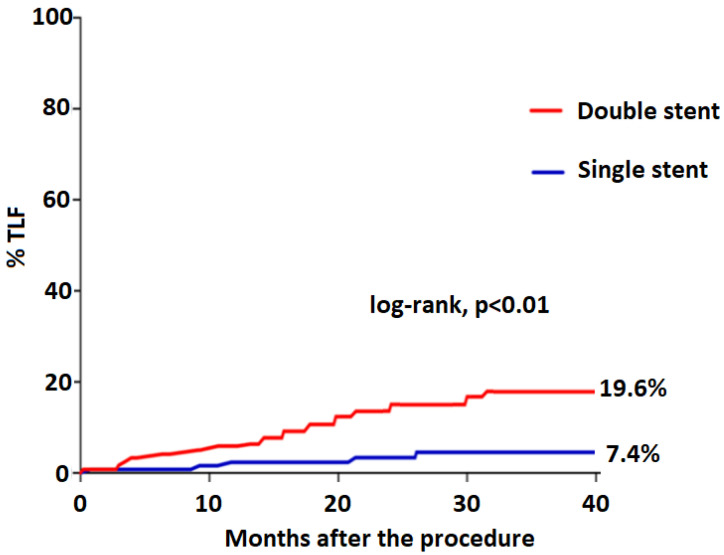
Kaplan–Meier analysis (log-rank) indicated that patients with a double stent had a high occurrence of TLF compared to those treated with a single stenting approach.

Clinical follow-up was available for all patients. At a median follow-up of 37.1 months (IQR 22.1–39.3), the TLF rate was 8.7% (n = 39): 5/155 (3.2%) in the provisional group; 10/53 (18.8%) in T/TAP group, 14/89 (15.7%) in the culotte group, and finally 10/148 (6.7%) in the NIT group of patients (*p* ANOVA = 0.14). The cardiovascular mortality rate was 2.9% (n = 13) while stent thrombosis was 0.89% (n = 4). Clinically driven angiographic follow-up was available in 135 patients (30.3%) at a mean time from the procedure of 7.8 ± 0.7 months and showed significant restenosis in 35 patients (clinically restenosis 7.9%), predominantly located at LCx ostium or within 5 mm from the ostium in 30 patients (85.7%) or in the LAD in the rest of 5 patients (14.2%). Patients with a double stent experienced TLF more frequently compared to those treated with single stenting (Figure 1).

Patients with dyslipidemia, severe calcifications requiring Rotablator use, renal failure, triple vessel disease, requiring multiple and long stents with high Syntax scores, had more TLF compared to those without such clinical, anatomic, and procedural characteristics (Table 5). On multivariate analysis, only dyslipidemia, Syntax score > 25, triple vessel disease, additional ostial or body lesions, and the use of Rotablator, resulted being an independent predictor of TLF (Table 6).

## 4. Discussion

Our retrospective study suggests that complex bifurcation LM PCI in NSTEMI clinical setting offers encouraging long-term clinical outcomes with low rates of TLF cardiovascular death and stent thrombosis either with single or double stent strategies, although the net rate of TLF appeared higher in single stent strategy. Among double stenting techniques, NIT appeared to have the safer profile in terms of TLF compared to Culotte and T/TAP techniques.

Improved stent technology and implantation techniques have made complex left main (LM) bifurcation percutaneous interventions (PCI) safer and more widely used as an alternative to standard aorto-coronary bypass surgery (CABG) in selected patients, especially in those in whom the surgical approach is refused or contraindicated [13,14,15,16]. As suggested by the latest recommendations of the European Bifurcation Club (EBC) [17] and accordingly to recent randomized clinical trials (RCT) and meta-analysis results [18], provisional stenting remains the gold-standard technique for the percutaneous interventional management of LM bifurcation disease. However, over the latest years, the role of double stenting techniques in distal LM bifurcation disease has gained increasing interest considering the positive results provided by different large analyses [19].

Although the results of the current literature body defined the single stent strategy with provisional stenting technique as the safest in terms of TLF in the general population [20], our study also suggests that, when faced with complex LM bifurcation disease, the double stent strategy offers acceptable results in terms of TLF, cardiovascular death, and stent thrombosis even in a challenging subset of patients, such as those with NSTEMI presentation. Regarding this, our retrospective analysis involving only patients with NSTEM presentation can fill up the gap in the last decade’s major trials and registries comparing PCI versus CABG in unprotected LM. These studies were not designed intentionally for NSTEMI patients: the proportion of NSTEMI patients in IRIS-MAIN, NOBLE, and EXCEL and studies ranged from 10 to 30% of the enrolled population, and no specific ad hoc analysis has been made for NSTEMI group of patients compared to the rest of the population [13,14,21].

In NSTEMI patients single stent strategy appeared to be slightly superior to double stenting: among double stenting technique, NIT resulted very similarly to provisional strategy despite the higher anatomical complexity as regards as TLF rate, explaining in part last trials of double versus single stenting in LM complex disease [22,23] which found a slight superiority of DK-crush over single stenting strategy. As already a previous study showed [24], NIT (which in the past was called nano-crush) resulted to have a very comfortable safety profile with the lowest rate of TLF compared to the well-known Culotte and T/TAP techniques. Advantages of such a technique are the very limited number of crushed struts which would improve, as already demonstrated by simulation studies [25], the rheologic profile at the bifurcation carina limiting the decrease of wall shear stress forces, usually attributable to heavy crush technique such as the mini-crush and Dk crush. Although the apparent worse outcomes for T/TAP and culotte compared to provisional and NIT did not reach a statistical significance due to global low numbers of events, it is likely that such differences in outcomes is clinically relevant.

Our study suffers from several limitations. Firstly, the retrospective non -randomized nature of the study was partially overcome by the size of the patients’ sample analyzed. Secondly, the absence of all the techniques which are in the spectrum of double stenting strategy, in particular the lack of use of DK-crush, which our domestic operators were not familiar with. Thirdly, the lack of matched comparison among different dual antiplatelet strategies in terms of both type and duration was difficult to evaluate due to multiple confounding factors. Finally, the different frequencies of IVUS imaging in a different subset of patients are driven by different case complexity, availability, and the operator’s discretion. In particular, the poor outcomes of T or TAP might be caused by a lower frequency of IVUS use in such cases compared to other subgroups treated with other techniques.

In conclusion, our retrospective study in NSTEMI patients with contraindications or refusal of surgery treated by either the single or double stent strategy resulted in low rates of TLF, cardiovascular death, and stent thrombosis in the long-term compared to literature results of LM PCI in non-acute settings. Ad hoc trials about different percutaneous strategies in NSTEMI patients are claimed to assess the real value of complex PCI compared to surgery in this clinical setting.

## Figures and Tables

**Table 1:** Demographic and clinical characteristics of the analysed cohorts of patients’ single versus double stent strategy.

	Single Stent N = 155	Double Stent N = 290	*p*
Age (years)	68.3 ± 9.1	70.4 ± 10.9	0.56
Male	82 (52.9)	159 (54.8)	0.85
Obesity	22 (14.2)	45 (15.5)	0.45
Arterial hypertension, n (%)	86 (55.5)	169 (58.2)	0.54
Dyslipidaemia, n (%)	63 (40.6)	132 (45.5)	0.04
Diabetes, n (%)	43 (27.7)	94 (32.4)	0.03
Previous smokers, n (%)	49 (31.6)	102 (35.1)	0.64
Active smokers, n (%)	28 (18.1)	45 (15.1)	0.68
Valvular heart disease, n (%)	33 (21.3)	71 (24.8)	0.79
LVEF (%)	52.5 ± 10.7	53.2 ± 9.8	0.77
CSS class	2.7 ± 1.1	2.5 ± 0.7	0.03
TIA/stroke, n (%)	41 (26.4)	90 (31.0)	0.71
eGFR < 60 mL/min/1.73 m^2^	24 (15.4)	48 (16.5)	0.88
HF, n (%)	54 (34.8)	96 (33.1)	0.91
COPD, n (%)	45 (29.0)	93 (32.0)	0.84
PAD, n (%)	38 (24.5)	59 (20.3)	0.12
EUROSCORE	20.3 ± 9.4	22.6 ± 6.4	0.03

LVEF: Left Ventricular ejection fraction; CCS: Canadian class score; TIA: Transient ischemic attack; HF: Heart failure; COPD: Chronic obstructive pulmonary disease; PAD: Peripheral artery disease; NIT vs. provisional stenting.

**Table 2:** Demographic and clinical characteristics of the analysed cohorts of patients divided for specific double stent technique.

	T or TAP N = 53	Culotte N = 89	NIT N = 148	*p*
Age (years)	69.1 ± 10.3	71.9 ± 11.7	70.3 ± 12.8	0.60
Male	29 (54.7)	45 (51.0)	85 (57.4)	0.55
Obesity	9 (16.9)	14 (15.7)	22 (14.8)	0.07
Arterial hypertension, n (%)	30 (56.6)	53 (59.5)	86 (58.1)	0.86
Dyslipidaemia, n (%)	23 (43.4)	41 (46.0)	68 (45.9)	0.74
Diabetes, n (%)	15 (28.3)	29 (32.6)	50 (33.7)	0.06
Previous smokers, n (%)	19 (36.0)	32 (35.9)	51 (34.4)	0.07
Active smokers, n (%)	9 (16.9)	13 (14.6)	23 (15.5)	0.09
Valvular heart disease, n (%)	14 (26.4)	21 (23.6)	36 (24.3)	0.84
LVEF (%)	54.1 ± 8.9	52.6 ± 10.1	53.1 ± 9.7	0.32
CSS class	2.4 ± 0.8	2.5 ± 0.9	2.6 ± 0.9	0.59
TIA/stroke, n (%)	16 (30.1)	29 (32.6)	45 (30.4)	0.78
eGFR < 60 mL/min/1.73 m^2^	9 (16.9)	14 (15.7)	25 (16.9)	0.82
HF, n (%)	18 (33.9)	27 (30.3)	51 (34.4)	0.85
COPD, n (%)	16 (30.1)	29 (32.6)	48 (32.4)	0.86
PAD, n (%)	11 (20.7)	16 (17.9)	32 (21.6)	0.84
EUROSCORE	20.2 ± 9.3	23.1 ± 6.5	24.5 ± 5.2	0.02

LVEF: Left Ventricular ejection fraction; CCS: Canadian class score; TIA: Transient ischemic attack; HF: Heart failure; COPD: Chronic obstructive pulmonary disease; PAD: Peripheral artery disease; NIT vs. provisional stenting.

**Table 3:** Lesions and procedural characteristics of the analysed cohorts of patients.

	Provisional N = 155	T or TAP N = 53	Culotte N = 89	NIT N = 148	*p* (ANOVA)
Three-vessel disease	88 (59.5)	30 (56.6)	61 (68.5)	113 (76.3)	0.03
LM lesion location					
Ostial, n (%)	24 (16.2)	9 (16.9)	15 (16.8)	32 (21.6)	0.67
Body shaft, n (%)	29 (19.6)	14 (26.4) **	33 (37.1)	60 (40.5) *	0.001
Distal LM, n (%)	155 (100)	53 (100)	89 (100)	148 (100.0)	0.99
Medina 1,1,1 bifurcation, n (%)	65 (43.9)	26 (49.0)	37 (41.6)	72 (48.6)	0.87
Medina 0,1,1 bifurcation, n (%)	44 (29.7)	15 (28.3)	27 (30.3)	38 (25.6)	0.56
Trifurcation, n (%)	46 (31.0)	12 (22.6)	25 (28.0)	38 (25.6) *	0.07
Calcification *, n (%)					
Moderate, n (%)	18/5 (10.5)	10 (18.8)	15 (16.8)	30 (20.2) *	0.09
Severe, n (%)	13 (8.7)	7 (13.2)	12 (13.4)	30 (20.2) *	0.25
Chronic total occlusion	32 (21.6)	8 (15.0)	12 (13.4)	28 (18.9)	0.12
LM, n	1	0	0	1	-
LAD, n	12	2	8	10	-
LCx, n	10	2	0	10	-
RCA, n	9	4	4	7	-
TIMI flow grade < 3					
Main vessel	15 (8.7)	5 (9.4)	6 (6.7)	10 (6.7)	0.57
Side branch	13 (10.5)	4 (7.5)	7 (7.8)	14 (9.4)	0.69
SYNTAX	28.8 ± 8.1	29.1 ± 7.6	30.3 ± 7.0	31.6 ± 6.3 *	0.02
FFR assessment					
LM, n	1	0	3	3	-
LAD, n	10	4	8	8	-
LCx, n	10	6	5	7	-
Stent characteristics					
Mean LM stent diameter (mm)	4.3 ± 0.8	4.3 ± 0.7	4.4 ± 0.8	4.5 ± 0.9	0.60
Mean number of stent	1.5 ± 0.5	2.2 ± 0.5	2.5 ± 0.5	2.8 ± 0.4	0.02
Global stent length (mm)	26.8 ± 10	33.8 ± 10	46.1 ± 11	46.4 ± 10	0.02
DAPT regimen					
Aspirin +Ticagrelor 12 months	50 (32.5)	18 (33.9)	32 (35.9)	48 (32.4)	0.72
Aspirin + Prasugrel 12 months	51 (32.9)	20 (37.7)	30 (33.7)	46 (31.0)	0.68
Aspirin + Clopidogrel 12 months	33 (21.3)	12 (22.6)	18 (20.2)	31 (20.9)	0.25
Aspirin + Ticagrelor/Prasugrel < 12 months	21 (13.5)	3 (5.6)	9 (10.1)	23 (15.4)	0.58

* Defined as moderate calcification (radiopaque densities noted only during the cardiac cycle and typically involving only 1 side of the vascular wall) or severe calcification (radiopaque densities noted without cardiac motion before contrast injection and generally involving both sides of the arterial wall). DAPT: double antiplatelet therapy; LAD: left anterior descending coronary artery; LCx: left circumflex coronary artery; LM: left main; RCA: right coronary artery. * *p* < 0.05 NIT vs. Provisional; ** *p* < 0.05 NIT vs. T or TAP.

**Table 4:** IVUS measurements in IVUS assessed patients.

1-Stent					2-Stent			
	Baseline		Post Stenting *		Baseline		Post-Stenting *	
	MLD (mm)	MLA (mm^2^)	MLD (mm)	CSA (mm^2^)	MLD (mm)	MLA (mm^2^)	MLD (mm)	CSA (mm^2^)
Mid LM	1.6 ± 1.2	2.6 ± 0.4	4.9 ± 0.6	14.8 ± 2.4	1.5 ± 1.2	2.6 ± 0.4	4.9 ± 0.5	14.8 ± 2.4
Distal LM	1.8 ± 1.4	2.5 ± 0.5	4.7 ± 0.2	13.9 ± 2.8	1.6 ± 1.2	2.2 ± 0.5	4.8 ± 0.3	14.0 ± 2.7
LAD ostium	1.3 ± 0.6	1.4 ± 0.5	3.7 ± 0.9	9.4 ± 0.4	1.3 ± 0.6	1.4 ± 0.4	3.6 ± 0.8	9.6 ± 0.4
LCx ostium	1.2 ± 0.9	1.2 ± 0.5	3.3 ± 0.8	9.2 ± 0.3	1.2 ± 0.9	1.2 ± 0.5	3.2 ± 0.9	9.1 ± 0.3

IVUS: Intravascular ultrasound. LAD: left anterior descending coronary artery; LCx: left circumflex coronary artery; LM: left main; MLD: minimal lumen diameter; MLA: minimal lumen area; CSA: Cross sectional area. * No significant difference in MLA and CSA between 1- and 2-stent strategies.

**Table 5:** Clinical, anatomical, and procedural parameters distribution among patients with and without TLF at 3 years of follow-up.

	With TLF N = 39 (%)	Without TLF N = 406 (%)	*p*
Gender (females)	11 (28.2)	193 (47.5)	0.02
Age ≥ 75 years	9 (23.1)	171 (42.1)	0.02
Obesity	4 (10.2)	69 (16.9)	0.28
Diabetes	15 (38.4)	125 (30.7)	0.32
Dyslipidemia	26 (66.6)	164 (40.4)	0.002
eGFR < 60 mL/min/1.73 m^2^	2 (5.1)	83 (20.4)	0.02
Triple vessel disease	35 (89.7)	260 (64.0)	0.001
Additional ostial LM lesion	33 (84.6)	50 (12.3)	<0.001
Additional body LM lesion	34 (87.1)	109 (26.8)	<0.001
Syntax > 25	37 (94.8)	301 (74.1)	0.004
Use of Rotablator	6 (15.3)	5 (1.2)	<0.001
Mean number of stent	2.8 ± 0.5	2.0 ± 0.5	0.02
Global stent length (mm)	33.7 ± 9.0	28.7 ± 8.1	0.06
IVUS	25 (64.1)	201 (49.5)	0.67
MACE	7 (17.9)	40 (9.8)	0.58
CV mortality	3 (7.6)	10 (2.4)	0.87

TLF: Target lesion failure; CV: cardiovascular; GFR: glomerular filtration rate; IVUS: Intravascular ultrasound; MACE: Major adverse cardiovascular event.

**Table 6:** Univariate and Multivariate Cox Regression analysis for TLF at three years of follow-up.

	Univariate			Multivariate		
	HR	95% CI	*p*	HR	95% CI	*p*
Gender (females)	1.25	0.87–1.44	0.65	-		
Age ≥ 75 years	1.16	1.02–1.29	0.04	-		
Obesity	1.32	0.84–1.22	0.38	-		
Diabetes	1.26	0.85–1.66	0.46	-		
Dyslipidemia	1.39	1.24–1.48	0.005	1.30	1.26–1.35	0.02
eGFR < 60 mL/min/1.73 m^2^	1.06	0.98–1.13	0.40	-		
Triple vessel disease	1.96	1.88–2.06	0.001	1.90	1.84–1.95	0.006
Additional ostial LM lesion	1.69	1.64–1.73	<0.001	1.62	1.58–1.65	<0.001
Additional body LM lesion	1.54	1.50–1.59	<0.001	1.46	1.41–1.49	<0.001
Syntax > 25	1.69	1.60–1.78	0.006	1.52	1.47–1.53	0.02
Use of Rotablator	1.48	1.44–1.50	<0.001	1.39	1.34–1.41	0.002
Mean number of stent	1.12	0.91–1.25	0.35	-		
Global stent length (mm)	1.02	0.92–1.06	0.09	-		

## Data Availability

The data are property of Rovigo General Hospital and protected by the Italian laws on privacy.
